# The Dynamics of Soybean Leaf and Shoot Apical Meristem Transcriptome Undergoing Floral Initiation Process

**DOI:** 10.1371/journal.pone.0065319

**Published:** 2013-06-06

**Authors:** Chui E. Wong, Mohan B. Singh, Prem L. Bhalla

**Affiliations:** Plant Molecular Biology and Biotechnology Group, ARC Centre of Excellence for Integrative Legume Research, Melbourne School of Land and Environment, The University of Melbourne, Parkville, Victoria, Australia; Wuhan University, China

## Abstract

Flowering process governs seed set and thus affects agricultural productivity. Soybean, a major legume crop, requires short-day photoperiod conditions for flowering. While leaf-derived signal(s) are essential for the photoperiod-induced floral initiation process at the shoot apical meristem, molecular events associated with early floral transition stages in either leaves or shoot apical meristems are not well understood. To provide novel insights into the molecular basis of floral initiation, RNA-Seq was used to characterize the soybean transcriptome of leaf and micro-dissected shoot apical meristem at different time points after short-day treatment. Shoot apical meristem expressed a higher number of transcripts in comparison to that of leaf highlighting greater diversity and abundance of transcripts expressed in the shoot apical meristem. A total of 2951 shoot apical meristem and 13,609 leaf sequences with significant profile changes during the time course examined were identified. Most changes in mRNA level occurred after 1short-day treatment. Transcripts involved in mediating responses to stimulus including hormones or in various metabolic processes represent the top enriched GO functional category for the SAM and leaf dataset, respectively. Transcripts associated with protein degradation were also significantly changing in leaf and SAM implicating their involvement in triggering the developmental switch. RNA-Seq analysis of shoot apical meristem and leaf from soybean undergoing floral transition reveal major reprogramming events in leaves and the SAM that point toward hormones gibberellins (GA) and cytokinin as key regulators in the production of systemic flowering signal(s) in leaves. These hormones may form part of the systemic signals in addition to the established florigen, *FLOWERING LOCUS T* (FT). Further, evidence is emerging that the conversion of shoot apical meristem to inflorescence meristem is linked with the interplay of auxin, cytokinin and GA creating a low cytokinin and high GA environment.

## Introduction

Flowering process governs seed set and hence affects agriculture productivity. Research carried out in understanding this fundamental process in crop species is thus vital in ensuring future food security under changing climate. Soybean [*Glycine max* (L.) Merrill] is the world’s largest source of oils and proteins and its capacity to fix atmospheric nitrogen through its symbiotic relationship with soil-borne microorganisms further enhances its significance in the world agriculture. As a paleopolyploid, soybean has undergone at least two major genome duplication and subsequent diploidization events resulting in a complex genome with homeolog expected for most genes [1]. It is a short-day plant grown broadly across the latitude but with each cultivar having a narrow range of north to south adaptation. This geographic adaptation of soybean is likely a result of genetic diversity associated with a large number of genes and quantitative trait loci regulating flowering behavior [2].

The floral initiation process is regulated by complex networks incorporating endogenous as well as exogenous cues in order to ensure the reproduction process occurring under optimal conditions. Studies carried out using *Arabidopsis thaliana*, a facultative long-day plant, have revealed the involvement of about 180 genes in controlling flowering time and a proportion of these genes occur in a network of six major flowering regulatory pathways [3].

The photoperiod and vernalization pathways regulate flowering in response to either seasonal changes in daylength or temperature while the ambient temperature pathway do so under the influence of daily growth temperature. The rest of the three pathways are more responsive to internal developmental cues and these involve the age, autonomous and gibberellins (GA) pathways. Central to these pathways are three floral pathway integrators: *FLOWERING LOCUS T* (*FT*), *SUPPRESSOR OF OVEREXPRESSION OF CONSTANS1* (*SOC1*/*AGL20*) and *LEAFY* (*LFY*) that are proposed to integrate signals from these multiple pathways and coordinate floral developmental program in the shoot apical meristem (SAM).

FT is the mobile flowering signal produced in leaves that travels to the SAM [4] and forms a complex with the bZIP transcription factor, FD. The FT/FD complex then induces the expression of SOC1, the first floral gene activated in the SAM after exposure to long-days converting the SAM into an inflorescence meristem (IM) [5], [6]. SOC1 activates *LFY* and similar to the FT/FD complex can also induce the expression of floral meristem identity genes such as *APETALA1* (*AP1*) triggering a developmental program culminating in the formation of flowers. Although the function of FT is conserved across different species [2], [7], [8], the fact that *ft* mutants are only late-flowering suggests additional factors could override the mutation eventually.

Counterparts of Arabidopsis flowering time genes are beginning to be studied in soybean [9] and the functional conservation of these orthologs has been demonstrated but with some intriguing variations. For example, while GmFT2b and GmFT5b are reported to have florigen-like functions like the Arabidopsis FT, they are repressed by the GmPHYA1 and GmPHYA2 under long-days and hence inhibit the flowering process [2]. This is in stark contrast to Arabidopsis whereby PHYA plays a promotive role together with CRY2 resulting in the stabilization of CONSTANS (CO), [10]. Furthermore, unlike Arabidopsis, it is the GmCYR1a and not GmCRY2a that play a role in promoting flowering [11].

We are interested in identifying transcriptional networks that contribute to the floral initiation process in soybean under inductive short-day. Our earlier studies have indicated the diversification of some gene expression and key regulators in shoot apical meristems of legume crops [12], [13], [14], [15]. While floral initiation ultimately involves the switch of developmental program at the SAM from leaf production to establishing floral meristems, the florigenic signal(s) are from the leaves that sense the change in the photoperiod. Accordingly, we used Illumina sequencing technologies to profile gene expression in the leaf as well as micro-dissected SAM in a time course experiment following short-day treatment. We explored the resulting dataset to identify major changes happening in the leaf or SAM leading to the activation of floral meristem identity genes.

## Materials and Methods

### Plant Materials and RNA Extraction

**Figure 1:**
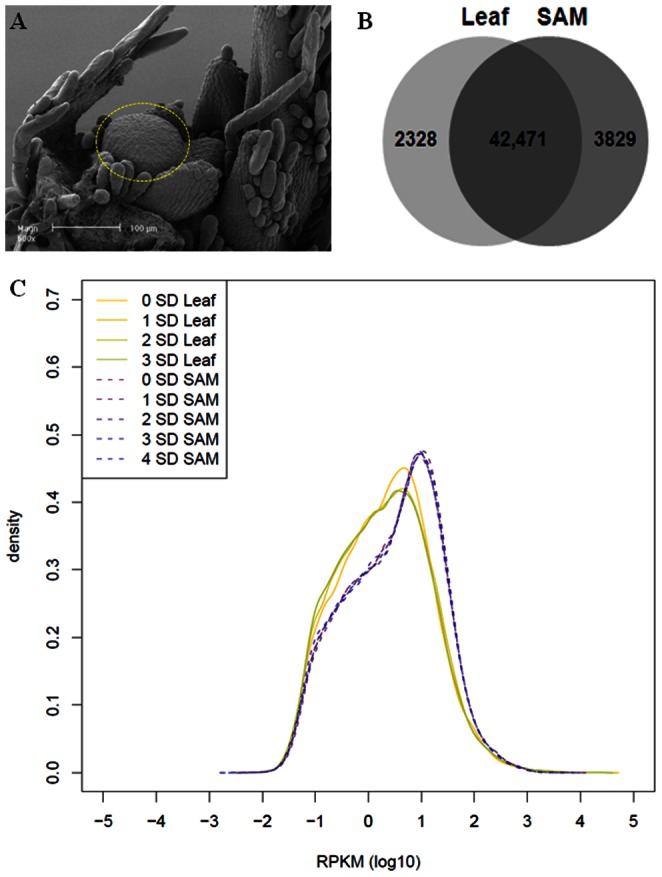
Expression of annotated transcripts. **A.** Soybean shoot apical meristem from 10 days old plant as viewed by SEM. Shoot apical meristem sample dissected is in dotted line. **B.** A Venn diagram showing the overlap of identified transcripts based on Glyma1 annotation for the leaf and SAM library. **C**. The expression level of transcripts in reads per kb per million (RPKM) for each library. SD, short-day.

Soybean plants [*Glycine max.* (L) Merr. Cv. Bragg] were grown from seeds in a greenhouse located at the University of Melbourne, Victoria, Australia for 10 days (0 short-day; plants with two fully expanded primary leaves) before being shifted to a growth chamber under a 10-hour light regime (150 µmol m-2 s-1) with a constant temperature of 25°C. A 26G syringe needle (Terumo Medical Corporation, NJ, USA) was used to dissect SAMs [12] from soybean shoot apexes with leaf primordia excluded as much as possible under the dissecting microscope at 40x magnification to create a meristem-enriched tissue collection (Fig. 1A). Approximately, 70 SAMs were dissected for each time point (0 short-day, 1 short-day, 2 short-day, 3 short-day and 4 short-day) while five leaves were randomly sampled from five individual plants for each time point (0 short-day, 1 short-day, 2 short-day and 3 short-day). All samples were collected within the first 3 hours of daylight. Dissected samples were quickly frozen in liquid nitrogen and stored at −80°C. Total RNA was extracted from dissected SAMs using Tri reagent (Sigma) according to manufacturer’s instructions and with DNAse digestion step incorporated.

### cDNA Library Preparation and Sequencing

Isolated RNAs were shipped on dry ice to Beijing Genomics Institute (China) for the subsequent cDNA library preparation and sequencing steps. The cDNA libraries were prepared according to the manufacturer’s instructions (Illumina). Briefly, poly(A) containing mRNA molecules were purified from 3 µg total RNA for each sample. The mRNA was fragmented before cDNA synthesis primed by random primers. The resulting cDNA was modified for subsequent adapter-ligation using Illumina PE adapters. These were then size-selected using agarose gel electrophoresis before being enriched with 15 rounds of PCR amplification. Nine pair-end libraries were constructed and sequenced according to the Illumina HiSeq™ 2000 platform sequencing protocols.

### Data Analysis

Raw sequence reads (Accession number SRP020868 (run accession number SRR824155-SRR824163) were filtered for low quality reads and trimmed off adapter sequence before being subjected to mapping analysis using SOAP2 [16]. The soybean genome sequence and annotated gene set available at Phytozome (www.phytozome.net) were used for the mapping and annotation. The abundance for each gene was calculated and expressed in RPKM [17]. A gene was considered differentially expressed during floral initiation in the leaf or SAM when it exhibited a significant difference in read abundance in at least one time point relative to the previous time point (L1-L0, L2-L1, L3-L2, S1-S0, S2-S1, S3-S2, S4-S3), which was tested using Audic-Claverie statistics [18] with the false discovery rate controlled at 0.1% [19].

## Results and Discussion

### Transcriptome Analysis of Soybean Leaf and SAM under Short-day

To capture the dynamics of mRNA expression changes in the leaf and SAM leading to the induction of floral meristem identity genes as a result of short-day treatment in soybean, transcriptome sequencing was performed. To this end, RNA were isolated from leaves and micro-dissected SAM, on 0 short-day (10-day-old soybean plants with two primary leaves), as well as at one day interval for the next three days after the plants were short-day treated. As our previous pilot study has identified the induction of a floral meristem identity gene (*GmAP1*) in the SAM on 4-short-day [20], an additional time point (4-short-day) was included for the SAM as a positive control for the induction of floral initiation process. In total, four leaf samples (0, 1, 2 and 3-short-day) and five SAM samples (0, 1, 2, 3 and 4-short-day) were sequenced using the Illumina HiSeq™ 2000 platform. The total number of 90-bp high quality pair-end reads generated was in excess of 200 millions with an average read of 26 millions per library (Table 1). Of these, approximately 82% on average could be mapped to the current first chromosome-scale assembly of the soybean genome (Glyma1) within which close to 69% with perfect match and the remaining mapped at less than or equal to 5bp mismatches (Table 1).

**Table 1:** Statistics of soybean transcriptome sequencing.

Average per library	Leaf	SAM
**Reads**	**26,757,451**	**26,583,770**
**Mapped reads**	**22,080,146 (82.5%)**	**21,761,333 (81.8%)**
**-Perfect match**	−14,855,994 (67.2%)	−15,248,666 70.1%)
**-Less than or equal to 5bp mismatches**	−7,224,152 (32.8%)	−6,512,667 (29.9%)
**Unmapped reads**	**4,677,305 (17.5%)**	**4,822,437 (18.2%)**

There is a total of 66,153 protein-coding loci predicted (Glyma1 annotation) and 48,623 of these were detected in at least one sample (73.5%) while the remaining (26.5%) were not expressed or expressed at a level too low to be detected in tissues examined (Table S1). These mapped reads are distributed evenly throughout the body of transcripts as revealed by the plot of number of reads relative to their position on cDNAs for each of the library (Figure S1). While 7.9% (3829) of the detected transcripts are specifically expressed in the SAM, 4.8% (2328) are found only in the leaf transcriptome (Figure 1A). On average, the SAM library seems to have a higher number of transcripts (36%) with greater than 90% gene coverage in comparison to that of leaf (27%) highlighting a greater diversity and abundance of transcripts expressed in the SAM (Figure 1).

**Figure 2:**
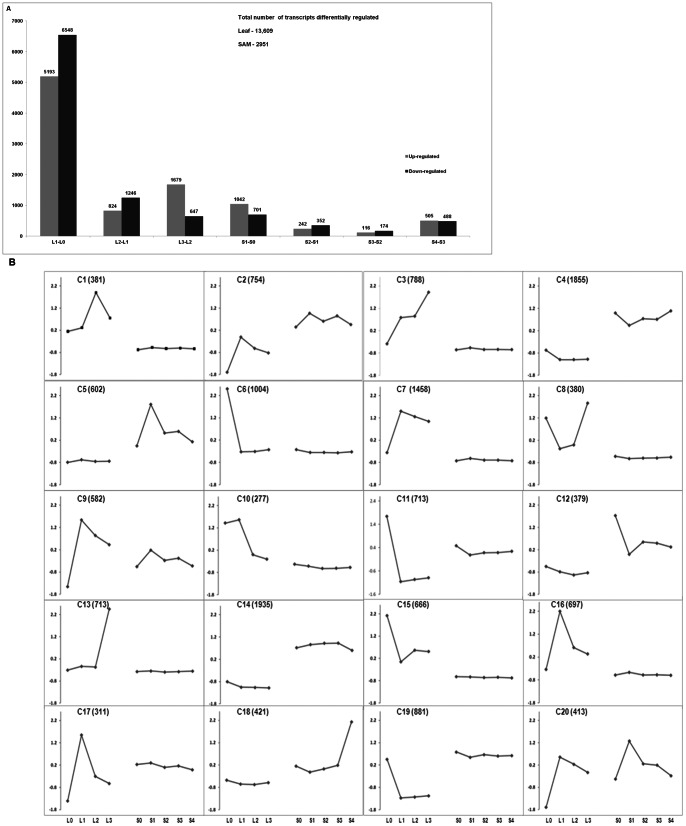
Differential expression of genes during short-day treatment. **A.** Number of genes significantly up-or down-regulated during the short-day treatment relative to the previous time point is indicated. **B.** K-means clustering illustrating the expression (Z-score normalized) profile of the soybean leaf and SAM transcriptome. Twenty clusters were used for a total of 15, 210 transcripts with significant expression profile changes in either leaf or SAM. Total number of transcripts in each cluster is indicated in parentheses. L0–L3: samples derived from leaf after 0-short-day (L0), 1-short-day (L1), 2-short-day (L2) or 3-short-day (L3) treatment. S0–S4: samples derived from SAM after 0-short-day (S0), 1-short-day (S1), 2-short-day (S2), 3-short-day (S3) or 4-short-day (S4) treatment.

Among the 48,623 transcripts, 13,253 (27%) were expressed below 1 read per kb per million (RPKM) reads in all samples and these were excluded from further analysis since a further down-regulation or up-regulation to below 1 RPKM level may not have significant effect on the developmental switch under study. To identify genes differentially regulated by the exposure to short-day and hence floral initiation process through transcriptome sequencing, we compared expression levels at consecutive time points using Audic-Claverie statistics [18] with false discovery rate [21] controlled at 0.1%. We identified 2951 sequences that were differentially expressed in the SAM while 13,609 genes with significant profile changes were uncovered from the leaf samples (Figure 2A). The transition to short-day caused the most perturbation in leaf transcriptome especially following 1short-day treatment as 11,741 transcripts were differentially regulated at 1short-day in the leaf in comparison to that on 0short-day (Figure 2A); representing close to 92% of the total number of sequences with significant profile changes detected in the leaf. Similarly, in the SAM, most changes in mRNA level occurred after 1short-day treatment with a total of 1743 sequences significantly differentially regulated following by the S4-S3 with a total of 993 sequences (Figure 2A). The later is likely a result of major reprogramming occurring in the SAM necessary for the floral initiation to occur.

### Functional Assessment of Differentially Expressed Transcripts

All transcripts with significant profile changes were then subjected to functional assessment by AgriGO [22] or Mapman [23]. The short-day exposure has the most effect on transcripts in the Plant Gene Ontology (GO) Slim term of “metabolic process’ and ‘response to stimulus’ in the leaf and SAM dataset, respectively (Table 2). The former consists of predominantly transcripts involved in primary metabolic processes (Table S2) reflecting necessary metabolic acclimation likely due to altered balance between carbon assimilation and utilization under shorter photoperiod. Sequences related to protein metabolic processes, in particular, proteolysis, are also represented in this category. These may be involved in triggering the rapid switch from one developmental program to another in response to the change in photoperiod since the significance of protein degradation driving developmental changes is well documented [24].

**Table 2:** Functional assessment of differentially expressed transcripts.

GO term	Description	Percentage	p-value
**A**			
GO:0009058	biosynthetic process	30.3	8.00E-08
GO:0050896	response to stimulus (hormone)	30.8 (9)	7.70E-08
GO:0019725	cellular homeostasis	2.3	8.30E-06
GO:0008361	regulation of cell size	3.2	1.50E-04
GO:0008152	metabolic process	60.1	1.60E-03
**B**			
GO:0050896	response to stimulus (hormone)	36.4 (10)	1.80E-11
GO:0010468	regulation of gene expression	17.2	1.20E-07
GO:0009058	biosynthetic process	32.8	1.30E-05
GO:0042592	homeostatic process	3.7	4.90E-05
GO:0009908	flower development	4.4	0.00031

Sequences that were differentially expressed in leaf (**A**) or SAM (**B**) during the short-day exposure were subjected to functional category assessment using AgriGO. A total of 11,939 (out of 13,609) or 2507 (out of 2951) sequences are successfully categorized and a representative of the top enriched GO terms for molecular processes (controlled at FDR <0.05) are shown. All functional categories identified are given in Table S2.

While transcripts involved in mediating responses to phytohormone are found within the ‘response to stimulus’ category, a great proportion of these are also stress-related sequences indicating general plant defense response mechanism were deployed when a change in photoperiod was perceived even in the absence of any biotic or abiotic threat. The category of ‘the regulation of cell size’ is another significantly enriched term for leaf dataset consisting of transcripts potentially involved in cell wall expansion and cell cycle progression (Table S2). As expected, sequences associated with ‘regulation of gene expression’ or ‘flower development’ are also among the top significant GO terms for the SAM dataset (Table 2).

### Induction of Flowering Time Regulators and Floral Meristem Identity Genes

**Figure 3:**
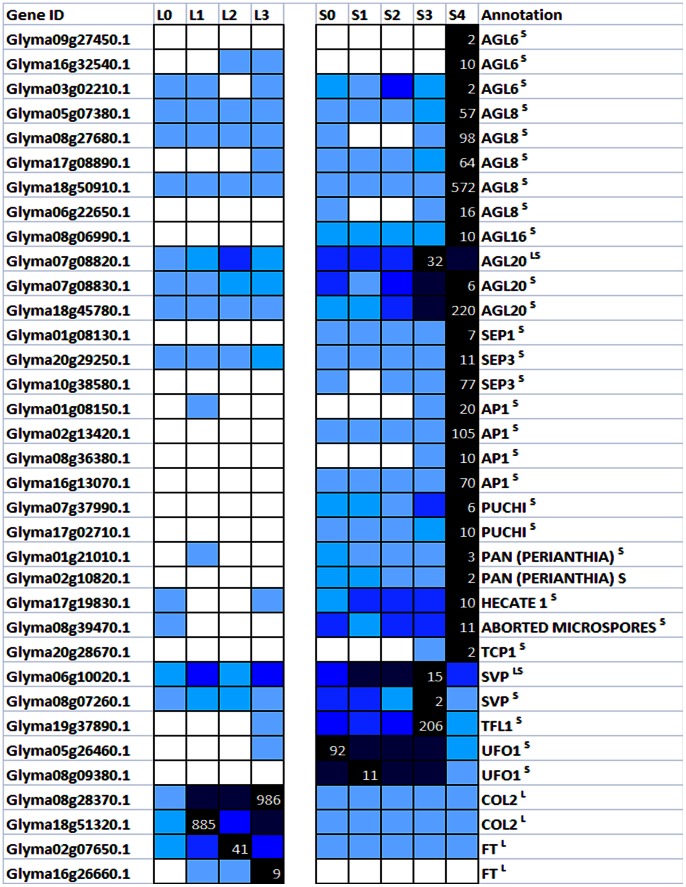
Expression of putative floral regulatory genes in soybean. Representatives of differentially expressed soybean transcripts predicted to encode various floral regulatory genes are shown. The highest expression level for each gene across different samples is given in RPKM value (see Materials and Methods). The level of expression for a gene across different samples are represented as percentage of the maximum expression level in colour code from 0% (white) to 100% (black). L0–L3: samples derived from leaf after 0-short-day (L0), 1-short-day (L1), 2-short-day (L2) or 3-short-day (L3) treatment. S0–S4: samples derived from SAM after 0-short-day (S0), 1-short-day (S1), 2-short-day (S2), 3-short-day (S3) or 4-short-day (S4) treatment. Transcripts with significant profile changes in the leaf (^L^) or SAM (^S^) or both dataset (^LS^) are indicated in the annotation.

To have an overview of the expression pattern of differentially expressed transcripts during short-day treatment in the leaf and SAM, these were grouped by their expression dynamics (Z-score normalized RPKM values) using K-means clustering algorithm into 20 clusters (Figure 2B). All homologs of previously characterized floral meristem regulators occur within Cluster 18 and these include *SOC1/AGL20*, *AGL8*/*FFUL*, *CAL*, *AP1*, PAN (PERIANTHIA) [25], *AGL6* [26] as well as a newly identified AP2/EREBP transcription factor gene, PUCHI [27] (Figure 3), indicating the conversion of the SAM to IM was initiated following 3 short-days treatment (Figure 3).

There are other floral regulatory genes within the same cluster including *ABORTED MICROSPORES* (Glyma08g39470.1), *SEPALLATA1* (Glyma01g08130.1), *SEPALLATA3* (Glyma20g29250.1), *TCP1* (Glyma20g28670.1) and *HECATE1* (Glyma17g19830.1). While *SEPALLATA1* and *SEPALLATA3* function as floral homeotic genes in Arabidopsis, *ABORTED MICROSPORES*, *HECATE1* and *TCP1* affect a much later process, the development of female reproductive tract [28], microspores [29] or floral organ symmetry growth [30]. Their expression levels were not as high as that of floral meristem identity transcripts and thus likely to increase at later stages of flower development. However, the earlier than expected up-regulation of these transcripts in the floral initiation process could contribute to the different floral developmental plan in soybean in comparison to Arabidopsis [31].

On the other hand, known floral repressors including *SHORT VEGETATIVE PHASE* (*SVP*) and *TERMINAL FLOWER1* (*TFL*) are found in Cluster 2 and Cluster 14, respectively and they were down-regulated on 4-short-day (Figure 3). GmTFL1b (Glyma19g37890.1) has recently been reported to control the stem growth habit of soybean [32] and in determinate cultivar such as Bragg used in this study, its level is expected to be rapidly down-regulated in the SAM for the transition to reproductive development to take place. Intriguingly, transcripts annotated as *UNUSUAL FLORAL ORGAN1* (UFO1) are also found in Cluster 14. In Arabidopsis, *UFO1* is an important counterpart to *LFY*’s action in promoting floral initiation process. The fact that it was not induced during the short-day treatment but down-regulated following the induction of other floral meristem identity genes on 4-short-day (Figure 3), implies a variation of the LFY/UFO network in legumes from that of Arabidopsis. Further evidence supporting this is the observation of unchanged transcription of *GmLFY* during the floral initiation process (Table S1), which is in contrast with Arabidopsis where *LFY* and *AP1* are consecutively activated [33].

As CO-FT module is key to the photoperiod pathway, we examined the corresponding orthologs in our leaf dataset. There are two close homologs of Arabidopsis CO in soybean and only one homoelog pair displayed rapid and sustained up-regulation of transcription following 1short-day treatment (Figure 3). As for FT, three soybean homologs could be found among the differentially expressed dataset (Glyma02g07650.1, Glyma16g04830.1, Glyma16g26660.1) but only two (Glyma02g07650.1, Glyma16g26660.1) displayed induced expression during the short-day exposure (Figure 3) suggesting these are likely the functional equivalent of the Arabidopsis FT. While Glyma16g26660.1 is previously annotated as GmFT2a and has the same function as Arabidopsis FT [2], Glyma02g07650.1 has not been reported as FT homolog. As the up-regulation of known floral meristem identity genes began on 3-short-day and thereafter, the flowering inducing signal from the leaf must have reached the SAM before 3-short-day. In other words, the generation of leaf derived floral signal have happened prior to the sampling on 3-short-day. The temporal expression of GmCO and GmFT is consistent with this hypothesis and furthermore the slightly later induction of GmFT than GmCO is in line with CO acting upstream of FT. The presence of known floral regulatory transcripts in our dataset confirms the appropriate response of the plants after exposure to short-day.

### Hormonal Events Associated with Floral Initiation

Environmental signals are known to directly affect hormonal levels in plants and therefore it is not surprising that sequences predicted to be hormone-related constitute a great proportion of transcripts with significant profile changes in both leaf and SAM datasets (Table 1). In fact, roles in flowering time control have been established for GA while there are also evidence implicating the roles of other hormones including cytokinin, ethylene, abscisic acid (ABA) and auxin [34] in controlling flowering time. We thus examined these transcripts further.

### Ethlyene and Abscisic Acid

Ethylene and ABA are universally known as stress hormones that inhibit growth with extensive crosstalk between the respective signaling pathways. In fact, there is a negative feedback regulatory loop between ABA and ethylene synthesis with ethylene reported to induce ABA synthesis while ABA inhibits the production of ethylene [35].

**Figure 4:**
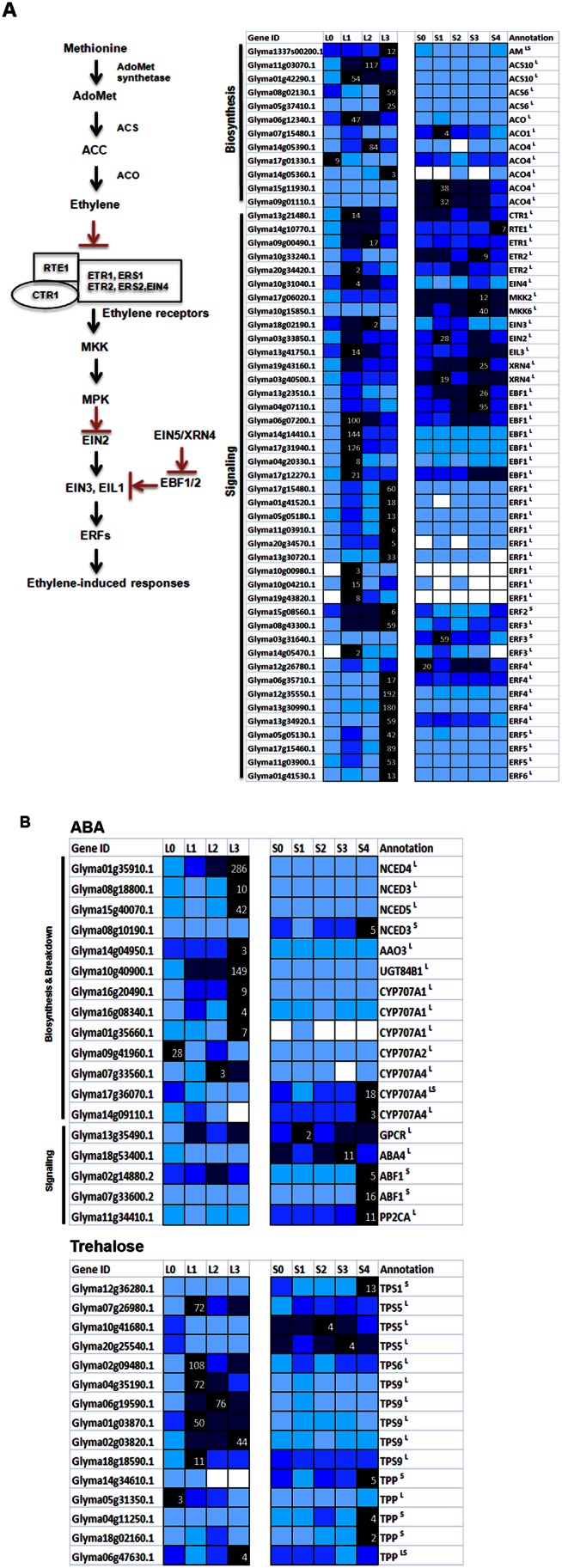
Expression of putative ethylene and ABA biosynthesis and signaling genes in soybean. **A**. A schematic diagram depicting ethylene biosynthesis and signaling [69] is given on the left while expression data of differentially expressed transcripts for respective annotated homologs are given on the right panel. Ethylene is perceived by repressing the action of receptor complexes including ETR/ERS/EIN4 receptors, RTE1, and Raf-like protein kinase CTR1, which negatively regulates downstream signaling component. When EIN2 is activated in the presence of ethylene, it stabilizes EIN3. EIN5/XRN4 regulates EBF1 (EIN3 BINDING F-BOX1) that degrades EIN3 in the absence of ethylene. **B**. Expression data of differentially expressed transcripts related to ABA (top) or trehalose (bottom) metabolism are shown. NCED, Nine-cis-epoxycarotenoid dioxygenase; AAO3, abscisic aldehyde oxidase3; UGT84B1, ABA glucosyltransferase; CYP707A, ABA hydroxylase; GPCR, G-protein coupled receptor for ABA; ABA4, ABA-deficient 4; ABF1, ABA-responsive element-binding factor1. Expression level is as in Figure 3.

**Figure 5:**
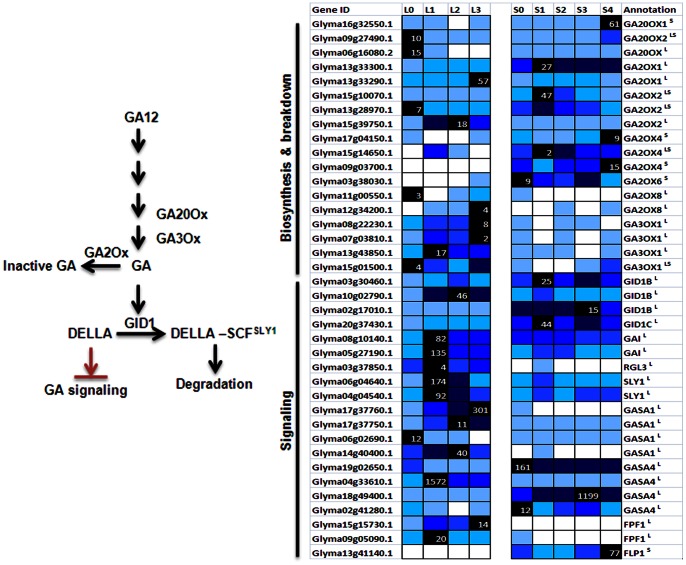
Expression of putative GA biosynthesis and signaling genes in soybean. GA biosynthesis and signaling mechanism represented in a simplified schematic diagram on the left [70] while expression data of differentially expressed transcripts for respective annotated homologs are given on the right. GA concentration is primarily regulated by the biosynthetic enzymes GA20Ox and GA3OX and GA-inactivating enzyme, GA2Ox. GID1 is a GA receptor and upon binding to GA, promotes an interaction between GID1 and DELLAs. This enhances the affinity between DELLAS and a specific SCF E3 ubiquitin ligase complex via SLY1, an F-box protein, which eventually promotes the degradation of DELLAs by the 26S proteasome. Expression level is as in Figure 3.

Ethylene biosynthetic transcripts as well as key signaling components are specifically enriched in the leaf dataset and these range from membrane receptors to protein kinases (Figure 4A). A great majority of these (49 out of 61) was up-regulated following 1short-day treatment while some at a later time point on 3short-day with one putative *ETHYLENE RESPONSE FACTOR1* (*ERF*1, Glyma10g04210.1) displaying the highest fold of increase (48-fold) on 1 short-day in comparison to 0 short-day. Ethylene has been long regarded as a growth inhibitor as well as a key coordinator of stress responses. The observation that many transcripts involved in coordinating cell expansion and cell division were down-regulated while stress-related sequences were up-regulated on 1-short-day in the leaf (Table S1) implicates ethylene having a role in regulating their expression. For instance, the growth arrest may be regulated by positive ethylene signaling since ethylene is known to inhibit growth in a DELLA-dependent manner [36] and there were markedly increased expression of these growth repressors (Glyma08g10140.1, Glyma05g27190.1, Glyma05g27190.1) in the leaf following 1short-day (Figure 5). The possible increase of ABA level in the leaf as indicated by the up-regulation of a number of ABA biosynthetic transcripts (Figure 4B) could also play similar roles in inhibiting growth and up-regulating stress-responsive transcripts (Table S1). Strikingly, there is also an increased expression of transcripts that break down ABA in the leaf dataset (Figure 4B) implying an autoregulatory mode for the maintenance of hormone homeostasis.

There is likely a feedback regulatory loop operating in the leaf for ethylene and ABA synthesis to prevent excessive growth inhibition resulting from the dual activity of these two hormones. However, the relevance of ethylene and ABA signaling in the leaf to floral initiation needs to be examined further especially since there is evidence demonstrating their floral inhibiting effects [35], [36]. For example, ethylene is known to inhibit flowering under abiotic stress partly via reducing endogenous GAs, a known floral promoter [36]. However, ethylene biosynthetic gene has also been identified as one of CO targets [6] and ABA can promote flowering in several short-day plants [37], [38] Perhaps both hormones function as a regulator in inhibiting growth in the leaf under shorter photoperiod that is necessary to accommodate for the increased sink strength of the shoot apex, which results in a better supply of assimilates to the SAM favoring the developmental switch. It is also possible that among the stress-related transcripts that are under the regulation of ethylene or ABA, there are some that could play additional roles in flowering initiation since there is report of the existence of a cluster of flowering control proteins in the interaction network associated with abiotic stress [39].

Meanwhile, as ABA signaling is linked with that of trehalose-6-phosphate (T6P) [40], [41], [42], we also examined the dataset for sequences related to T6P metabolism (Figure 5). Trehalose is a disaccharide that is produced as a result of the activity of two enzymes, trehalose phosphate synthase (TPS) that catalyses the transfer of glucose from uridine diphosphate glucose to glucose-6-phosphate producing trehalose-6-phosphate (T6P), and trehalose-6-phosphatase (TPP) that hydrolyses T6P to release trehalose. It is believed that the phoshorylated form of trehalose, T6P, is the signaling component that regulates its effect for example on sugar signaling, vegetative growth as well as floral transition process in Arabidopsis [42], [43], [44].

Putative *TPS* and *TPP* transcripts were represented in both leaf and SAM dataset. For the leaf dataset, there were seven putative *TPS* transcripts that were up-regulated after 1 short-day (fold change ranging from 2 to 12) and only two putative *TPPs* with significant expression changes but down-regulated on 1 short-day, in contrast to *TPS*s (Figure 5). The high expression of *TPS* after 1short-day was maintained throughout the time course investigated. There is thus a possible sustained increase of T6P level in the leaf under short-day, which could interfere with carbon allocation and growth in a mechanism involving ABA metabolism. While T6P may serve the primary role of coordinating the metabolic shift in the leaf under short-day photoperiod, it could also play an important role in initiating flowering process as *tps1* mutant are unable to flower [43]. The temporal profile of *TPS* and *TPP* transcripts in the SAM markedly differs from that of the leaf with increased expression detected only on 4short-day coinciding with the induction of floral meristem identity genes such as *GmAP1*. *TPP* gene is linked to inflorescent branching in maize presumably through the alteration of trehalose level [45]. The increased expression of trehalose-related sequences on 4short-day in the SAM is consistent with this function.

Our previous study has reported an increase in ABA level at the SAM during the floral initiation process [20], [46]. The up-regulation of transcripts in ABA biosynthesis and signaling were also observed in the SAM in this study (Figure 5). As ABA has been reported to affect the expression of trehalose metabolism genes [47], ABA could thus modulate the expression of the corresponding sequences in the SAM although these transcriptional changes could also be influenced by sugar [44].

### Gibberellic Acid and Short-day Treatment

The level of bioactive GA species is regulated by the final biosynthetic reactions and inactivation steps catalyzed by GA 20-oxidases (GA20Ox) and GA 3-oxidases (GA3Ox), and GA 2-oxidases (GA2Ox), respectively [48]. The changes of the expression level of the biosynthetic transcripts indicate an increase of GA level in both the leaf and SAM during the floral initiation process (Figure 5). In the leaf, putative *GmGA3Oxs* showed overall markedly increased expression following 1short-day while it was the expression of *GmGA20Ox* (Glyma09g27490.1, Glyma16g32550.1) that was up-regulated significantly from 3short-day onwards in the SAM (Figure 5). This may reflect biosynthetic steps catalyzed by GmGA20Ox represent the key control point for regulating GA level in the SAM while *Gm3Ox* for the leaf during the switch from vegetative to floral development. In fact, it has been shown that the expression of any GA biosynthetic gene may increase or decrease depending on the physiological response [49]. Furthermore, the over-expression of either gene has been effective in increasing GA level in transgenic plants [50].

Intriguingly, the expression profile of a GA-inactivating enzyme, GmGA2Ox (Glyma17g04150.1) is very similar to that of GmGA20Ox (Glyma09g27490.1, Glyma16g32550.1) and there are also a few GmGA2Ox that were up-regulated on 1-short-day preceding that of GmGA20Ox in the SAM (Figure 5). The induction of both biosynthesis and deactivating genes alludes to the maintenance of hormone homeostasis being autoregulatory as in the case of ABA. Moreover, as the SAM sampled in this study consists of a diverse array of cell types and as the synthesis and perception of hormonal signals are often cell- and tissue-specific, it is possible that while certain cell types may experience activated GA signaling, others may face the opposite. Perhaps the up-regulation of GA2Ox seen here is necessary to restrict the access of GA to certain cells such as the stem cells as low GA regime is required to maintain their meristematic activities [51]. Similar observation of the up-regulation of positive and negative components of the GA metabolism pathway has also been reported in a microarray study investigating the Arabidopsis floral meristem development [52].

GA could be part of the systemic signals that initiates flowering as GA has been shown to move from the leaf to the shoot apex with the endogenous GA in the shoot apex increasing up to 100 fold prior to floral initiation in Arabidopsis under non-inductive short-day [53]. The induction of GA biosynthesis transcripts observed in the leaf in this study is consistent with this role. As GA is known to stimulate phloem loading [54] and florigen such as FT is phloem-borne [4], it is conceivable that GA could play additional role other than directly activating the expression of the floral integrator *SOC1* in the SAM by facilitating the transport of FT from the leaf vasculature to the SAM.

On the other hand, FLOWERING PROMOTOING FACTOR1 (FPF1) was proposed to mediate between GAs and the regulation of flowering time [55] *FPF1* is up-regulated in the SAM at the transition to flowering [55] but the corresponding soybean orthologs (Glyma15g15730.1, Glyma09g05090.1) are expressed only in the leaf suggesting a diverged function in soybean. Its roles may have taken over by a FPF1-like gene (FLP1, Glyma13g41140.1) with its transcript exclusively found in the SAM and significantly up-regulated on 4short-day (Figure 5). FLP1 could thus form part of the floral inducing network in the SAM responding to GA stimulus.

### Auxin and the Floral Initiation Process

**Figure 6:**
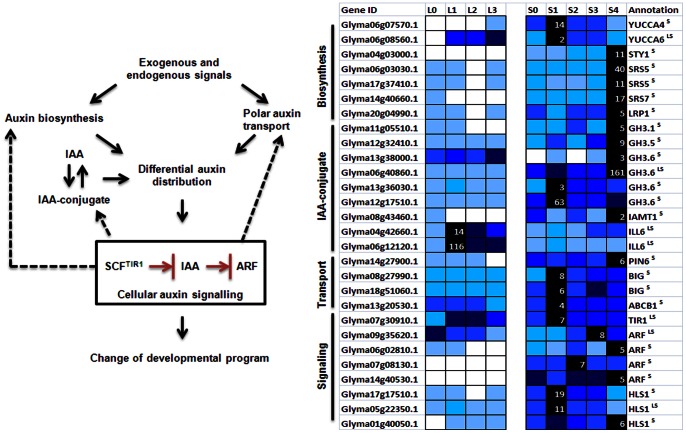
Expression of putative auxin-related genes in soybean. An overview of auxin regulatory mechanism is given on the left with possible feedback regulations indicated by dashed arrows [56] while expression data of differentially expressed transcripts for respective annotated homologs are given in the right panel. In the nucleus, IAA binds to an F box protein called TIR1 and stabilizes the interaction between TIR1 and transcriptional repressors AUX/IAA proteins, targeting them for proteolysis. STY1, SRS5, SRS5, SRS7 and LRP1 are all members of SHI family of transcription factors with STY1 known to stimulate auxin biosynthesis in Arabidopsis [57]. Expression level is as in Figure 3.

IAA is the most abundantly occurring form of auxin and its level is influenced by its biosynthesis, transport and the hydrolysis or formation of IAA conjugates (Figure 6). The pathways for auxin biosynthesis are yet to be fully elucidated but members of the YUCCA family of flavin monooxygenase-like enzymes have recently been reported to catalyze the rate-limiting step for auxin biosynthesis through a tryptophan-dependent pathway [56].

Soybean transcripts related to auxin metabolism, transport and signaling are expressed in the SAM during vegetative development (Figure 6). This is expected since one of the main sites of auxin synthesis is within the shoot apex containing young actively growing tissues. Among sequences that were clustered together with known reported floral meristem identity genes such as AP1 (Cluster 18) are putative orthologs of SHI (SHORT INTERNODES) family of transcription factors reported to be positive regulators of auxin biosynthesis [57], a number of auxin response regulators as well as those involved with maintaining auxin homeostasis by conjugating IAA to amino acid or methyl ester (Figure 6). Enzymes that catalyze the conjugation of auxin to amino acid such as GH3s are auxin-inducible and thus proposed to be part of a negative feedback mechanism in preserving cellular auxin homeostasis [56]. It is noteworthy that several other auxin-related genes have different expression dynamics with rapid up-regulation following 1short-day. These include putative *GmYUCCA4* (Glyma06g07570), GmYUCCA6 (Glyma06g08560.1), IAA-amino acid conjugate hydrolase (Glyma06g12120.1) and transcripts involved in polar auxin transport (Figure 6). There is thus likely an increase in cellular auxin level resulting from the increased auxin biosynthesis, release of free active auxin from the conjugate or the redistribution of auxin to create an auxin gradient. This could subsequently trigger the up-regulation of auxin responsive transcripts as seen on 4short-day coincident with the induction of *GmAP1*. Auxin is critical for flowering process as it defines the site of flower initiation, controls floral organ growth and patterning as well as subsequent events determining reproductive fitness [56]. Our study has nevertheless implicated an early role of auxin in the floral initiation process prior to the induction of floral meristem identity genes.

### Cytokinin and Short-day Induced Flowering

**Figure 7:**
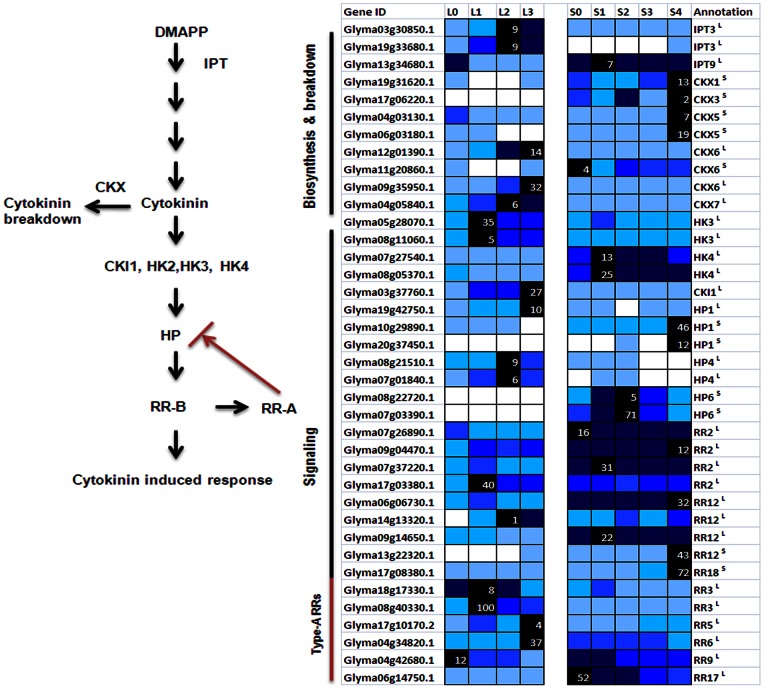
Expression of putative cytokinin biosynthesis and signaling genes in soybean. The rate-limiting step of cytokinin biosynthesis is catalyzed by *ATP/ADP-ISOPENTENYLTRASNFERASE* (IPT) gene family as indicated by the schematic diagram [60] on the left. Transmembrane histidine kinases (HK3, HK4) are cytokinin receptors that are auto-phosphorylated following the initial cytokinin perception. These then transfer the phosphate group to members of the HISTIDINE PHOSPHOTRANSFER proteins (HP1, 4, 6) family. HPs subsequently translocate to the nucleus to phosphorylate the RESPONSE REGULATORs (ARRs) proteins of either type-A or type-B. Activated type-A and type-B RRs negatively and positively regulate cytokinin signaling, respectively. Expression level is as in Figure 3.

There is an overall increase of transcripts related to cytokinin biosynthesis and signaling that can be interpreted as an increase in the hormone level in the leaf following 1short-day (Figure 7). Sequences annotated as *CYTOKININ OXIDASE* (Glyma12g01390.1, Glyma11g20860.1, Glyma09g35950.1, Glyma04g05840.1) that degrades cytokinin were also significantly up-regulated suggesting there is a likely attenuation or enhancement of cytokinin signaling depending on the cell type. The possible increase of cytokinin level in the leaf may precede the induction of floral meristem identity genes in the SAM. An increased level of cytokinin has been found first in the leaf extracts followed by the SAM of Arabidopsis plants induced to flower by a single long-day and the application of cytokinin has also been found to promote flowering in Arabidopsis under short-days [58], [59]. It is therefore tempting to speculate that the initial heightened cytokinin-mediated signaling is to orchestrate the network necessary to bring about the transport of flowering signals including cytokinin to the SAM. Furthermore, while there was no significant up-regulation of cytokinin biosynthesis gene in the SAM (Figure 7), there was an up-regulation of several cytokinin signaling components suggesting a possible contribution of leaf-derived cytokinin to the increase of cytokinin level in the SAM leading to the up-regulation of these sequences. The cytokinin signaling components activated in the SAM that are of particular interest include a putative *HISTIDINE PHOSPHOTRANSFER PROTEIN 6* (HP6) homeolog pair and B-type response regulators (RR12, Glyma13g22320.1; RR18, Glyma17g08380.1) that are positive regulators in the cytokinin signaling circuitry. While *GmHP6s* (Glyma08g22720.1, Glyma07g03390.1) show exclusive expression in the SAM with an increased expression on 1-short-day that peaked at 2-short-day and subsequently down-regulated, *GmRR12* and *GmRR18* showed similar expression dynamics to that of *GmSOC1* with at least 3-fold induction in the SAM on 3-short-day indicating they can be novel regulator in the SAM during the floral initiation process (Figures 3 & 7). As HPs are proposed to mediate signaling between the plasma membrane–bound cytokinin receptors and the response regulators within the nucleus [60], the timing of the up-regulation of *GmHP6* followed by *GmRR12* and *GmRR18* implicates *GmHP6* likely act upstream of these response regulators.

There was a delay in the increased expression of putative *GmCKX*s in the SAM as compared to the leaf and this can be interpreted as to allow for an initial accumulation of cytokinin in the SAM as a result of the transport from leaves. Cytokinin has been shown to induce auxin biosynthesis as well as its homeostatic mechanisms in the root tip [61]. Increases in transcript abundance on 1short-day for similar transcripts are also observed in the SAM (data not shown) suggesting the leaf-derived cytokinin could be behind the proposed rise of auxin level in the SAM. However, this also means leaf-derived signals must reach the SAM before the sampling on 1-short-day. There was a rapid increase in the expression of putative *GmIPT3s* (Glyma03g30850.1, Glyma19g33680.1) in the leaf on 1-short-day with the expression level peaking on 3-short-day. It is possible that the up-regulation of *GmIPT3*s observed in the leaf is still timely for cytokinin to play its part in inducing auxin biosynthesis in the SAM. This is interesting in light of recent reports that auxin can induce the expression of CKX and hence cytokinin breakdown [62]. An intuitively obvious mechanism for the induction of several GmCKXs as seen in this study is via auxin especially since there is correlative evidence suggesting an increase of auxin level in the SAM on 1short-day. It may be inferred from all these that there is a feedback regulation of cytokinin level in the SAM involving auxin.

A closer look of these GmCKxs expression profiles indicates that they could be functionally differentiated by their expression patterns (Figure 7). *GmCKX6* and *GmCKX7* were up-regulated in the leaf while it was the *GmCKX1*, *GmCKX3* and *GmCKX5* in the SAM. The Arabidopsis *CKX3* and *CKX5* have recently been reported to be expressed in the centre of the IM and procambium of the IM, respectively with the double *ckx3ckx5* mutant forming larger inflorescence and floral meristems due to increased cytokinin level and hence delaying cellular differentiation [59]. Cytokinin is known to inhibit GA biosynthesis [63] and the up-regulation of GmCKXs observed in this study more or less coincided with that of GA biosynthesis transcripts (Figures 5 & 7). The later up-regulation of *GmCKX* may thus decrease cytokinin level in the SAM to allow for proper cellular differentiation as well as the accumulation of GA which could take over its role in activating *SOC1* in concert with FT, and subsequently a plethora of floral meristem identity genes as seen on 4short-day. However, the observation that Arabidopsis *ckx3ckx5* double mutants undergoes rather normal floral developmental process with only effect on size of the floral meristems [59] suggest that there are redundancy in the regulatory network increasing GA level necessary for the activation of floral developmental program. For instance the GmCKX1 that has similar expression profile as the GmCKX3and GmCKX5 as seen in this study could perform similar and thus redundant roles in Arabidopsis and furthermore there are reports that auxin can directly stimulate GA biosynthesis in a range of plant species including the monocot barley [64].

### Short-day Responsive Processes and Hormonal Regulation of Floral Initiation

**Figure 8:**
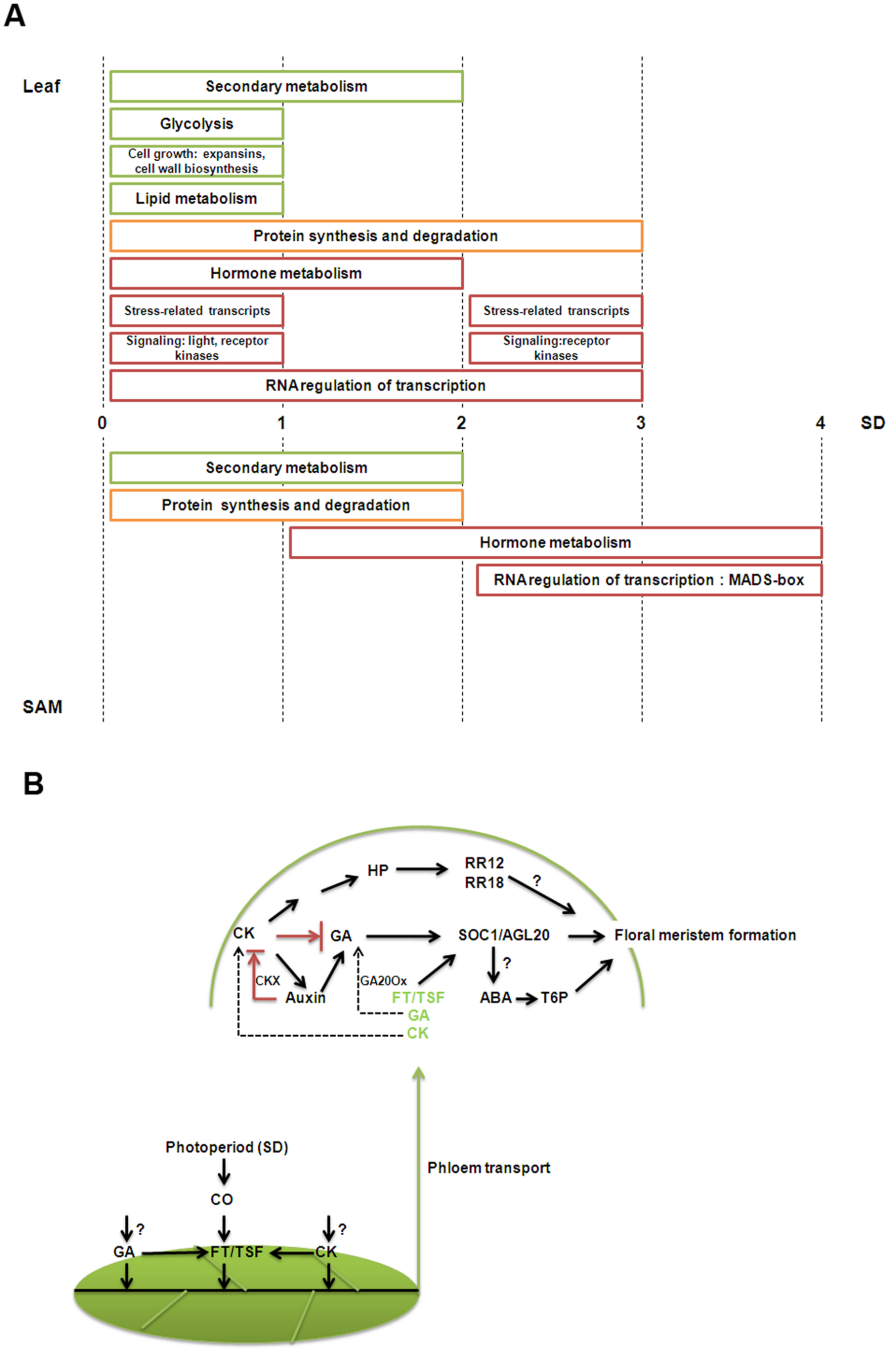
The floral initiation process in soybean. **A.** An overview of short-day responsive processes in leaf and SAM of soybean. Functional groups of transcripts largely up- or down- regulated are depicted schematically as red or green boxes, respectively while those containing both up- or down-regulated sequences are represented in yellow box. A complete list of Mapman significantly enriched functional categories is given in Table S2. **B.** A hypothetical model illustrating potential molecular events underlying the floral initiation process in soybean with an emphasis on hormonal regulation. The model shows the action of auxin, cytokinin (CK) and GA in concert with other known factors (described in Results and Discussion) in regulating the developmental transition in soybean. In particular, GA and CK may form part of the systemic signals in addition to FT. The induction of CKX possibly via auxin to breakdown cytokinin is likely to create a low cytokinin and high GA environment triggering the conversion of SAM to inflorescence meristem.

Representatives of significantly enriched functional groups as deduced from Mapman and AgriGO analysis over the time course of the short-day treatment is summarized in Figure 8A. Rapid sensing and signaling of the change in photoperiod likely involves various leaf transcripts in ‘signaling’ and ‘transcription factor’ categories. As sugar is known not only as fuels but also as signaling molecules, sequences in carbohydrate metabolism could also serve similar signaling roles. In response to the signaling events, major reprogramming occurs in both leaves and the SAM as evident by a great number of transcripts differentially expressed in the ‘protein synthesis and degradation’ category. This ultimately leads to the induction of various MADS-box transcription factors in the SAM from 3-short-day onwards.

Hormones may play predominant roles in the short-day-induced floral initiation process (Figure 8B). Both GA and cytokinin have been shown to induce flowering via FT or its paralogue, TSF [65], [66], [67]. Our hypothesis that they could be additional factors as florigenic signals is consistent with the fact that *ft* mutants are only late-flowering [4]. Once in the SAM, GA or cytokinin may exert their floral promoting roles by directly activating *GmSOC1* as well as contributing to a low cytokinin and high GA environment. The balance of cytokinin and GA (high cytokinin and low GA) is known to be critical in the maintenance of vegetative meristem homeostasis [60]. Our transcriptome analysis however alludes to low cytokinin and high GA level being critical for the conversion of vegetative meristem to IM. Multiple mechanisms are potentially in place to result in such hormonal regime as there is likely an influx of GA from the leaf as well as increased local production in the SAM that give rise to increased GA level. The induction of CKX possibly via auxin to breakdown cytokinin is to reinforce the low cytokinin to GA ratio especially since there is also a strong likelihood of cytokinin import to the SAM from the leaf under short-days. Interactions among these hormones form the basis in generating a specific signal to the SAM to eventuate in flowering. Meanwhile, germination is another developmental transition that responds to similar environmental cues as flowering and a recent study has unraveled common elements shared between genetic pathways regulating these two transitions [68]. As both ABA and GA are known to play roles in regulating seed germination and GA is known to regulate flowering, it is not unprecedented that our study has consistently implicated ABA roles in the floral initiation process.

The results described here highlight the utility of a genomics-based approach in enhancing our understanding of a vital developmental process, floral initiation. Our study has provided essential frameworks to investigate further the molecular network underlying the developmental transition.

## Supporting Information

Figure S1 **Distribution of mapped reads in relation to transcripts’ body.**
(PDF)

Table S1 Transcriptome of soybean leaf and shoot apical meristem undergoing short-day induced floral initiation process.
(XLSB)

Table S2 Functional assessment of differentially expressed transcripts by AgriGO or Mapman.
(XLSX)
