# Supplementary figures and images for: The Dynamics of Soybean Leaf and Shoot Apical Meristem Transcriptome Undergoing Floral Initiation Process
Source: PLoS One. 2013 Jun 6;8(6):e65319. doi: 10.1371/journal.pone.0065319 (PMC3675103; doi:10.1371/journal.pone.0065319)

Supplemental Figure 1. Distribution of mapped reads in relation to transcripts' body

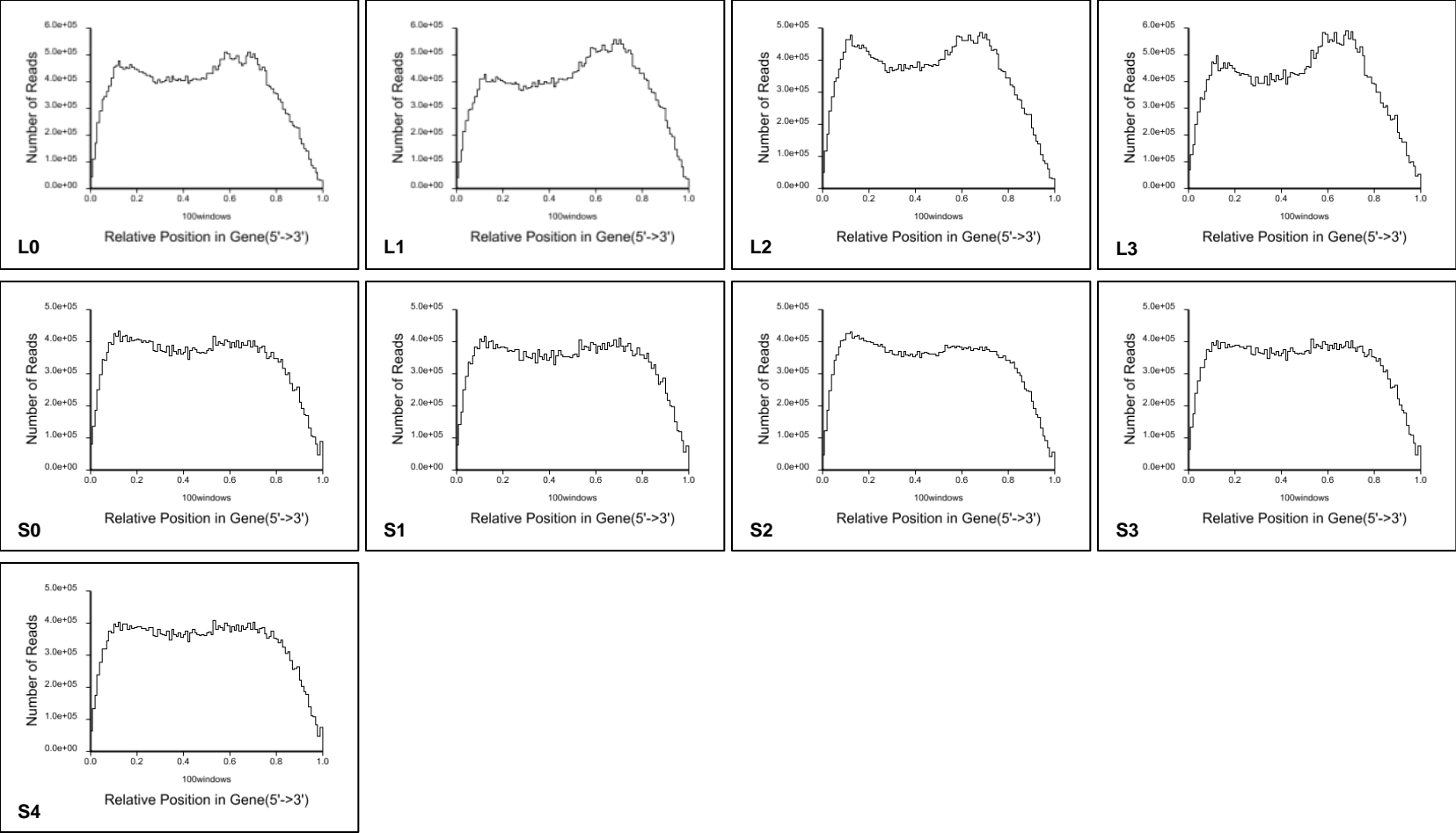

Supplement: Figure S1 — Distribution of mapped reads in relation to transcripts’ body. (PDF) [file pone.0065319.s001.pdf]
